# Strigolactones as a hormonal hub for the acclimation and priming to environmental stress in plants

**DOI:** 10.1111/pce.14461

**Published:** 2022-10-21

**Authors:** Marta Trasoletti, Ivan Visentin, Eva Campo, Andrea Schubert, Francesca Cardinale

**Affiliations:** ^1^ DISAFA, PlantStressLab Turin University Turin Italy

**Keywords:** abiotic stress, acclimation, avoidance, escape, strigolactones, tolerance

## Abstract

Strigolactones are phytohormones with many attributed roles in development, and more recently in responses to environmental stress. We will review evidence of the latter in the frame of the classic distinction among the three main stress acclimation strategies (i.e., avoidance, tolerance and escape), by taking osmotic stress in its several facets as a non‐exclusive case study. The picture we will sketch is that of a hormonal family playing important roles in each of the mechanisms tested so far, and influencing as well the build‐up of environmental memory through priming. Thus, strigolactones appear to be backstage operators rather than frontstage players, setting the tune of acclimation responses by fitting them to the plant individual history of stress experience.

## Summary statement

Strigolactones act as backstage operators rather than frontstage players in stress acclimation strategies (avoidance, tolerance, possibly, escape), by tuning them to the plant individual history of stress experience.

## 1 INTRODUCTION

Strigolactones are a family of plant hormones with a very diversified set of functions. First discovered as root‐exuded germination signals for parasitic plants (Cook et al., 1966), they were later assigned their first beneficial role as branching factors for arbuscular mycorrhizal (AM) fungi (Akiyama et al., 2005) and thus, as indirect facilitators of mineral nutrition. In 2008, they were found to be the long‐sought branching inhibitor hormone for which the synthesis or perception was compromised in distinct sets of mutants: *more axillary growth* (*max*) in *Arabidopsis thaliana* (arabidopsis); *dwarf*/*high tillering dwarf* (*d/htd*) in *Oryza sativa* (rice); *ramosus* (*rms*) in *Pisum sativum* (pea); and *decreased apical dominance* (*dad*) in *Petunia* × *hybrida* (petunia) (reviewed in Al‐Babili & Bouwmeester, 2015). More functions have been added in the following years, namely for root development and plasticity (reviewed by Mitra et al., 2021). Shortly thereafter, it became clear that strigolactones are involved in the response to abiotic stresses, first of all, nutrient deficiency and more recently, osmotic stress among others (Andreo‐Jimenez et al., 2015; Cardinale et al., 2018).

Among the many possible environmental constraints to plant growth, drought is one of the main ones (Bodner et al., 2015). Indeed, low water availability is a challenging condition for all living cells; understanding to what extent organisms can ‘learn’ to cope with drought stress episodes remains a fascinating question in basic biology that plants are optimally suited to answer. To loosely categorize, there are three types of strategies traditionally identified, which plants can adopt to increase their chance of survival in the face of low water availability: avoidance, tolerance and escape (Levitt, 1980). Different plants may have evolved priority for individual strategies, but still have the whole set of response strategies available, which can be co‐opted to a range of abiotic stresses. Broadly speaking thus, these categories can depict responses to all environmental stresses and will be used here as a more general paradigm for acclimation strategies in plants.

The first strategy is avoidance, which is achieved by optimizing water uptake by roots (in the so‐called ‘water spenders’), or by increasing water use efficiency and conservation by closing stomata and thickening the leaf epidermis and cuticle (in ‘water savers’). The second is tolerance, which is conspicuously acquired via the mitigation of stress impact by structural and biochemical mechanisms. The third is escape, which is mainly achieved by altering the pace of the developmental programme as to shorten the life cycle, and flower shortly after drought recovery. It must be emphasized that the boundaries between these schematic paths may be blurred, in that plants may adopt a combination of strategies depending on the species, but also on the intensity and the speed of stress onset (severe vs. mild, sudden vs. gradual), which affects the ability of the plant to acclimate to the incoming stress (e.g., Morabito et al., 2022; Ruehr et al., 2019). Also, the previous stress experience of the plant—be it of the same or different nature—steers the plant response towards a strategy that it would not necessarily favour if stress naïve. Building an ‘environmental memory’ can thus help the plant to better acclimate to its specific, fluctuating environment. Indeed, in the most common situation, the first period of sub‐lethal stress can prepare (prime) the plant for a subsequent (challenging) stress either similar or dissimilar in nature, thus triggering a form of plant memory (Bäurle, 2018; Kinoshita & Seki, 2014; H. Liu, Able, et al., 2022).

In the frame of such classic interpretation of plant responses to stress, this review will focus on the most recently reported evidence of strigolactone contribution to stress avoidance, tolerance and escape. The picture we will try to sketch is that of a hormonal family playing important roles in most aspects of environmental stress acclimation mechanisms tested so far, influencing as well the build‐up of environmental memory through priming.

## 2 LATEST DEVELOPMENTS IN THE SYNTHESIS, PERCEPTION AND TRANSPORT OF STRIGOLACTONES

A detailed review of current knowledge of strigolactone synthesis up to 2021 can be found in (Yoneyama & Brewer, 2021), while perception, signalling and transport are excellently covered by (Mashiguchi et al., 2021), to which the reader is directed. For the purpose of this review, the main molecular players will be outlined concisely and a few more recent findings, not reported in the above reviews, will be highlighted.

### 2.1 Biosynthesis

**Figure 1:**
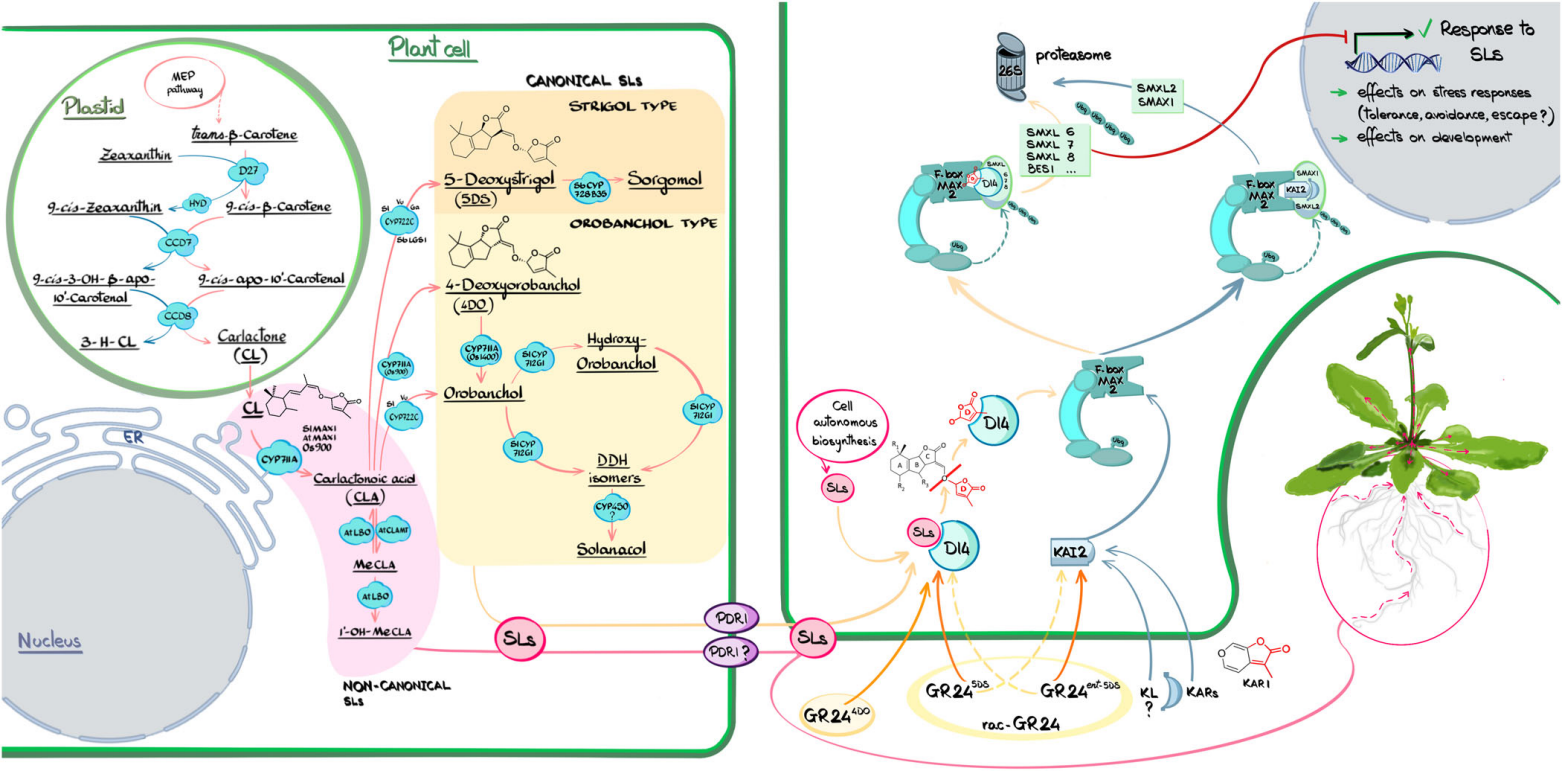
The strigolactone (SL) biosynthetic (cell on the left) and signalling pathways (on the right). Catabolic steps are not represented, as well as exceptions to the pictured transduction pathway (see text for details). KAR1 is pictured as a representative of the karrikin family. CCD, CAROTENOID CLEAVAGE DIOXYGENASE; CL, carlactone; CLA, carlactonoic acid; CLAMT, CLA methyl transferase; D, DWARF; DDH, didehydro; 4DO, 4‐deoxy‐orobanchol; 5DS, 5‐deoxy‐strigol; HYD, HYDROXYLASE; KAI2, KARRIKIN INSENSITIVE2; KARs, karrikins; KL, KAI2 ligand; GGPP, geranyl geranyl diphosphate; LBO, LATERAL BRANCHING OXIDASE; LGS1, LOW GERMINATION STIMULANT1; MAX, MORE AXILLARY GROWTH; MeCLA, methyl CLA; PDR1, PLEIOTROPIC DRUG RESISTANCE1; SMAX1, SUPPRESSOR OF MAX2 1; SMXLs, SMAX1‐LIKEs.

Strigolactones are a large family of compounds currently comprising over 30 members and growing. They can be subdivided into canonical strigolactones (containing an ABC‐ring connected via an enol‐ether bridge to a methylbutenolide d‐ring) and non‐canonical ones, in which the A, B or C rings are missing. A further distinction is between strigol‐ or orobanchol‐type strigolactones, depending on the orientation of the C ring (Yoneyama & Brewer, 2021); with few exceptions, individual plant species produce a blend of either type. Strigolactone synthesis starts in plastids, by the sequential action of three conserved enzymes on all‐*trans*‐β‐carotene to produce the key intermediate carlactone, a compound sharing with strigolactones the number of C atoms and the presence of a butenolide ring. Such core enzymes are, in biosynthetic order, the carotenoid isomerase DWARF27 (D27) and the carotenoid cleavage dioxygenases (CCD) 7 (MAX3/D17/HTD1/RMS5/DAD3) and CCD8 (MAX4, D10, RMS1 and DAD1) (Yoneyama & Brewer, 2021). Additionally, as shown in rice, the core biosynthetic module (D27, D10, D1) can also act on a hydroxylated derivative of all‐*trans*‐β carotene (zeaxanthin) leading to hydroxylated carlactone, which may in turn act as the precursor of as‐yet unidentified strigolactones (Baz et al., 2021). After carlactone exits the plastid, the pathway diversifies and more than one biosynthetic options are available in different species. In arabidopsis, the CYP711A MAX1 (Abe et al., 2014; Seto et al., 2014), CARLACTONOIC ACID METHYLTRANSFERASE (CLAMT) (Mashiguchi et al., 2022; Wakabayashi et al., 2021) and LATERAL BRANCHING OXIDASE (LBO) (Brewer et al., 2016) cooperate to produce respectively the non‐canonical strigolactones carlactonoic acid, methyl carlactonoate and hydroxymethyl carlactonoate, even though there is still uncertainty on the fine details of LBO activity (Mashiguchi et al., 2022) (Figure 1 left).

Beyond arabidopsis, the pathway downstream of carlactone to generate different structures of canonical strigolactones may entail, for example, several paralogues of the CYP711A MAX1 cooperating sequentially, as in rice (Yoneyama et al., 2018; Y. Zhang et al., 2014). It may also recruit, downstream of MAX1 orthologues, other CYP450 enzymes such as CYP722C isoforms in cotton (*Gossypium arboretum*), tomato (*Solanum lycopersicum*) and cowpea (*Vigna unguiculata*) (Wakabayashi et al., 2019, 2020), or the CYP728B35 LOW GERMINATION STIMULANT1 (LGS1) in sorghum (*Sorghum bicolour*) (Gobena et al., 2017; Wu & Li, 2021). The CYP712G1 enzyme of tomato was very recently shown to oxidize orobanchol to didehydro‐orobanchol isomers, one of which can be further converted to solanacol (Y. Wang, Durairaj, et al., 2022). Finally, one last important piece of the biosynthetic jigsaw has been recently identified in the form of the first catabolic enzyme for strigolactones, named CARBOXYLESTERASE15 (AtCXE15) in arabidopsis. This enzyme, and its orthologues in other tested species, can degrade both canonical and non‐canonical strigolactones; AtCXE15 and, possibly, paralogues in this large family such as AtCXE20 or others are important in fine‐tuning the effects of strigolactones on shoot branching (Xu et al., 2021) and stress (Roesler et al., 2021).

### 2.2 Perception and signalling

Also our understanding of strigolactones perception and signalling has progressed fast. With respect to the core signalling pathway, elucidated several years ago (Mashiguchi et al., 2021), the main advancements have come from new hypotheses on the origins of strigolactone signalling in land plants, and from a better molecular understanding of the perception and early transduction steps.

Strigolactone perception starts by physical binding to the receptor D14 (DAD2/RMS3), an α/β‐fold hydrolase protein which retains enzymatic activity but with extremely low turnover. This implies that the hormone is rather slowly hydrolysed to a tricyclic ABC and a d‐ring moiety (Hamiaux et al., 2012). Initially, it was postulated that ligand hydrolysis is necessary for strigolactone signalling (De Saint Germain et al., 2016; R. Yao et al., 2016); however, to accommodate some inconsistencies (Carlsson et al., 2018; Seto et al., 2019), hydrolysis‐independent signalling mechanisms have been proposed more recently.

It must be emphasized that there is not yet complete consensus on the finer details, especially regarding the sequential changes in molecular conformations and the role of the covalent modifications of D14 needed to achieve optimal sensitivity (Bürger & Chory, 2020; Mashiguchi et al., 2021). Nonetheless, according to one of the most recent interpretations, what triggers initial signalling is not the covalent binding of a hydrolytic by‐product of strigolactones in the active site of D14, but rather the destabilization of the latter upon physical interaction with the intact ligand. Once the D14‐strigolactone complex is formed, D14 destabilization makes it competent to bind the F–box protein MAX2 (D3/RMS4) (Zhao et al., 2015), a key transducer in the signalling cascade. MAX2–D14–strigolactone can then recruit a member of the D53/SMXLs (SUPPRESSOR OF MAX2 1‐LIKE) family, and subsequently mediate its polyubiquitination (Figure 1 right). Additional intramolecular changes within D14 upon strigolactone hydrolysis, and in MAX2, allow the release of ubiquitinated D53/SMXLs to the proteasome, their degradation and thus, transcriptional de‐repression of strigolactone‐responsive genes (Tal et al., 2022). It has been shown that among the members of the SMXL family, SMXL6/7/8 are responsible for the repression of most strigolactone signalling under regular conditions in arabidopsis (L. Wang, Wang, et al., 2020) (Figure 1 right). It is notable that SMXL6 also acts as a transcription factor targeting its own gene, among many others (Tang & Chu, 2020; L. Wang, Wang, et al., 2020). Other MAX2 interactors have been identified: one of them is *bri1*‐EMS‐suppressor 1 (BES1), which was initially characterized as a positive regulator in the brassinosteroid signalling pathway and later proposed as a regulator of strigolactone‐dependent shoot branching (Y. Wang et al., 2013). Another is, in rice, the DELLA suppressor of gibberellin signalling, SLENDER RICE1 (SLR1) (Nakamura et al., 2013), and although the relevance of the latter interactions for shoot branching inhibition seems dubious (Bennett et al., 2016), these partners may indeed act in different contexts and affect hormonal cross‐talk under specific conditions. Following the removal of D53/SMXLs, D14 becomes accessible to further ubiquitination and subsequent proteasomal degradation, enabling feedback regulation of the signalling cascade (Chevalier et al., 2014; Tal et al., 2022). Compound to ligand hydrolysis and persistent receptor occupation by the ligand, this step may gradually desensitize those tissues in which strigolactone signalling becomes intense (Chevalier et al., 2014; Seto et al., 2019).

D14 receptors include members that have undergone wide differentiation in plants and beyond. A recent study found a D14‐like protein in the phytopathogen *Cryphonectria parasitica* that interacts with strigolactones, hydrolyses them and is needed for their perception, even though the ecological significance of strigolactones for this organism is unresolved (Fiorilli et al., 2022). Also the perception of strigolactones as germination stimulants in seeds of parasitic plants relies on an expanded family of D14‐like receptors, called HYPOSENSITIVE TO LIGHT (HTL). *HTL* genes turned out to be present in all seed plants but with a possible different role than D14 proper. The first *HTL* gene discovered in arabidopsis was called *KARRIKIN INSENSITIVE2* (*KAI2*) because its mutant, while not affected in strigolactone perception, is insensitive to compounds named karrikins. These are abiotic in origin, as they are smoke components produced by combustion of plant tissues; they act as germination stimulants for fire‐succession species and structurally share a butenolide ring with strigolactones (Waters et al., 2012). However, while strigolactones have a butenolide ring with a methyl group that is essential for bioactivity, the corresponding methyl group of karrikins is nonessential for perception by KAI2, which indeed seems to prefer desmethyl butenolides (J. Yao et al., 2021). Perception of karrikins seems to have no significant biological role in arabidopsis, so KAI2 is thought to perceive primarily an endogenous and yet unknown molecule tentatively named KAI2 ligand; its structure is expected to share structural resemblance to karrikins, at least in its essential features (Conn & Nelson, 2016) (Figure 1 right).

The analogies and intersections between the two sibling pathways, either triggered by strigolactones or KAI2 ligand/karrikins, are not limited to the ligand and receptor (Q. Wang, Smith, et al., 2022). MAX2 is used by both (Nelson et al., 2011), and this realization has forced a revaluation of all initial attributions to strigolactones of *max2* phenotypes. The fact that most of the early works made use of a racemic mixture of the strigolactone analogue GR24 (*rac*‐GR24) to describe the effects of exogenous strigolactones further obfuscated the picture, since it contains two stereoisomers, GR24^5DS^ and GR24^*ent*‐5DS^ (Figure 1 right). The latter, albeit carrying an unnatural stereoconfiguration, stimulates both D14 and KAI2 to a certain extent, at least in arabidopsis (Scaffidi et al., 2014; L. Wang, Wang, et al., 2020). The former instead, which has a 5‐deoxy‐strigol configuration, seems to be more specific for D14—even though under certain conditions, it may be perceived weakly also by KAI2 (Villaecija Aguilar et al., 2019). More recently, it was found that one of the two other possible GR24 stereoisomers, GR24^4DO^ (which reflects the deoxy‐orobanchol configuration and is normally not present in commercial *rac*‐GR24 mixtures) is less potent than GR24^5DS^ but more specific to D14 stimulation in arabidopsis (L. Wang, Wang, et al., 2020) (Figure 1 right). This highlights how care should be exerted in attributing pharmacological findings to either pathway. For this same reason, recent works trying to untangle this knot have made use of pathway‐specific mutants coupled to more specific ligands, for example, karrikins vs GR24^5DS^ (Scaffidi et al., 2014), karrikins vs GR24^4DO^ (L. Wang, Wang, et al., 2020), or GR24^*ent*‐5DS^ vs GR24^4DO^ (L. Wang, Xu, et al., 2020) to stimulate KAI2 versus D14, respectively. Additionally, proteins that are clear KAI2 paralogues from their primary sequences can exhibit divergent ligand stereoselectivity and even have affinity for strigolactone‐type molecules, as in the case of KAI2A and KAI2B in pea (Guercio et al., 2022), making in silico‐only functional predictions less reliable than in other cases.

The evolutionary origins of strigolactone and KAI2 ligand/karrikin signalling components are being investigated actively, in an effort to pinpoint, among the many effects of this molecular family, which is ancestral and which derived. The presence of the core biosynthetic pathway (D27, CCD7, CCD8) in green algae, before the evolutionary appearance of obvious perception and signalling components, rather suggests an exogenous role for ancestral strigolactones. This hypothesis is strongly supported by the recent identification of the novel strigolactone molecule bryosymbiol in the bryophyte *Marchantia paleacea*, which lacks the ability to perceive it, but uses it to attract mycorrhizal partners in the rhizosphere (Kodama et al., 2022). With respect to the strigolactone versus KAI2 ligand/karrikin perception pathways, initial hypotheses had placed KAI2 as ancestral to D14 (Waters et al., 2012). The complete pathway seems to have been achieved in bryophytes after gradual acquisition of KAI2, MAX2 and SMXLs (Delaux et al., 2012; Q. Wang, Smith, et al., 2022). In turn, phylogenetic analyses suggest that plant KAI2 was derived from bacterial RsbQ via horizontal gene transfer before the emergence of streptophytes (Q. Wang, Smith, et al., 2022), while D14 was derived, in seed plants, from the neofunctionalisation of KAI2 paralogues (Bythell‐Douglas et al., 2017; Waters et al., 2012).

Current knowledge of the origins, structure and roles of individual members in the SMXL protein family have been excellently reviewed by (Temmerman et al., 2022), who also outline the most promising research avenues to clarify their functioning. A big scientific effort is underway to attribute transducer roles of D14 and KAI2 signals to individual members of the D53/SMXL family in arabidopsis. As a rule of thumb, it seems that SUPPRESSOR OF MAX2 1 (SMAX1) and SMXL2 tend to transduce events downstream of KAI2 signalling, while SMXL6/7/8 are in charge of the D14‐initiated pathway (Temmerman et al., 2022) (Figure 1 right). Nonetheless, some exceptions are already being reported. For example, SMXL6/7/8 have been linked to root skewing downstream of KAI2, together with SMAX1/SMXL2 in one report (Swarbreck et al., 2019) while not in a following one that carefully dissected the KAI2 vs D14‐dependent root phenotypes (Villaecija Aguilar et al., 2019). GR24^4DO^ could stimulate degradation of SMXL2 specifically and unexpectedly downstream of D14 (L. Wang, Wang, et al., 2020; L. Wang, Xu, et al., 2020), and D14/SCF^MAX2^/SMAX1 complexes could be successfully co‐precipitated in vivo (Q. Li et al., 2022). On the other hand, strigolactone‐related mutants do not phenocopy SMAX1 overexpression, at least under regular conditions, so it is possible that such interactions only happen when strigolactone concentrations increase under specific environmental conditions, for example, stress (see below).

### 2.3 Transport

Regarding strigolactone transport, we refer the reader to recent reviews such as Mashiguchi et al. (2021), and only wish to mention here the recent identification and characterization of orthologues of the petunia protein PDR1 (PLEIOTROPIC DRUG RESISTANCE 1), the first membrane transporter shown to be involved both in strigolactone exudation in the rhizosphere, and in cell‐to‐cell strigolactone movement (Kretzschmar et al., 2012; Sasse et al., 2015). PDR1 is a member of the ancient and ubiquitous ATP‐binding cassette (ABC) protein family and belongs to the ABCG subfamily, which is the most numerous in plants. The relatively high similarity among the many family members has slowed down the transfer of results from *Petunia* spp to other species; the recent functional characterization of PDR1 orthologues in *Medicago truncatula* and tomato is a welcome advancement (Banasiak et al., 2020; Bari et al., 2021). It is important to notice that at least in arabidopsis, carlactone, carlactonoic acid and methyl carlactonoic acid are actively translocated from root to shoot via PDR1, and may be themselves the main bioactive strigolactones in this species (Mashiguchi et al., 2022). It is unclear, for plant species that produce also canonical strigolactones, whether only mature strigolactones are transported systemically via PDR1, and/or their precursors. The hypothesis that there might be two different routes of transport in the shoot has also been advanced, one to distribute locally synthetized strigolactones to adjacent tissues, and one to transport them across a long distance (Borghi et al., 2016) (Figure 1 right).

## 3 STRIGOLACTONES AND STRESS RESPONSES: THE OSMOTIC STRESS CASE STUDY

Several recent reviews have covered the growing amount of evidence pointing to a role for strigolactones in abiotic stress responses. This wealth of data started with the finding that strigolactone synthesis is increased in roots facing low nutrient availability, in particular phosphorus and nitrogen (reviewed in Andreo‐Jimenez et al., 2015 and not touched upon here). Since 2014, the contribution of strigolactones to osmotic stress responses has been rather extensively documented as well. We will try to highlight their role in both avoidance and tolerance‐based acclimation strategies to abiotic stress, with non‐exclusive emphasis on osmotic stress.

### 3.1 The model for osmotic stress avoidance: Organ‐specific strigolactone dynamics

**Figure 2:**
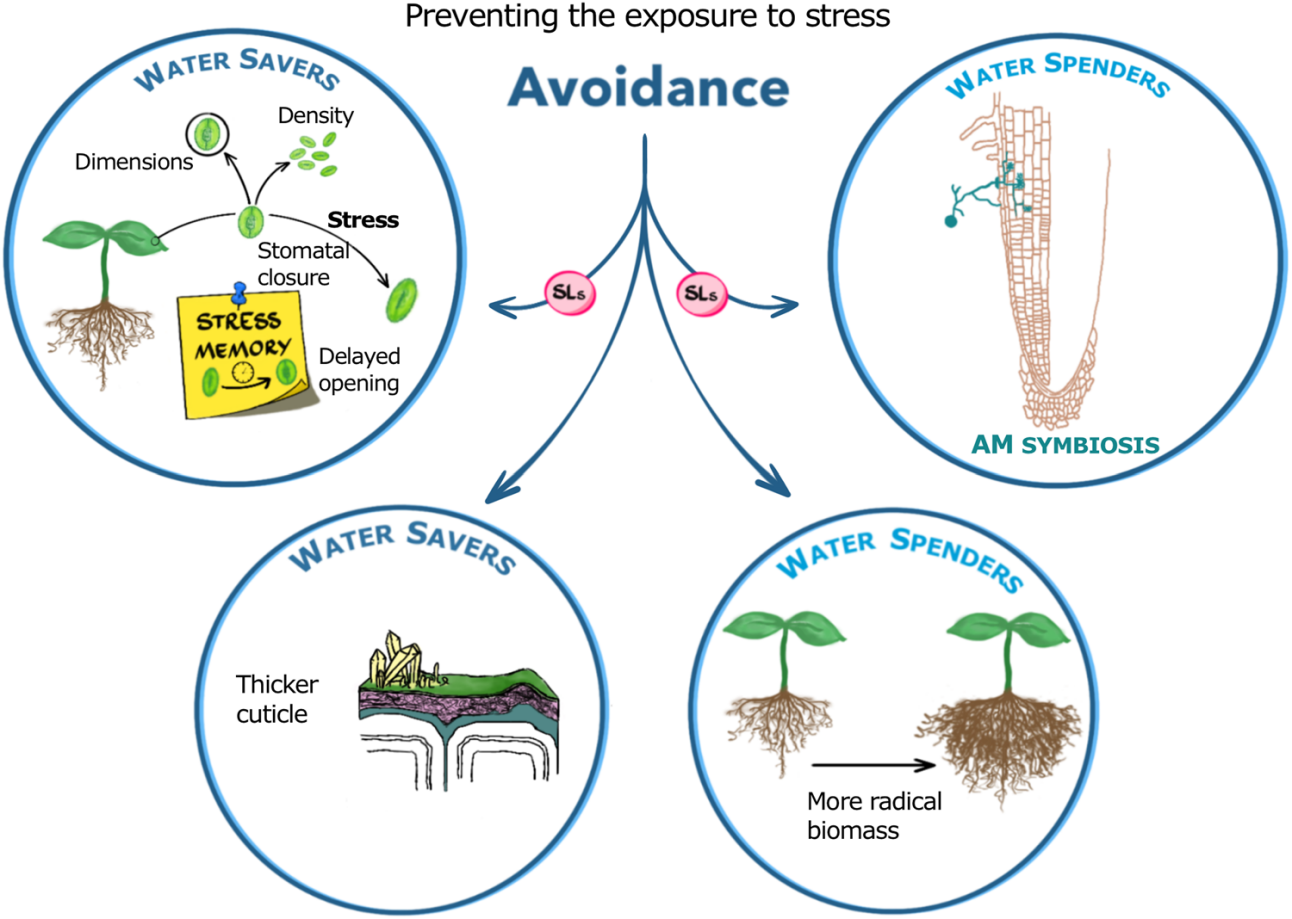
The influence of strigolactones (SLs) on the main water‐spending and water‐saving mechanisms in drought avoidance. Strigolactones support water savings by promoting ABA‐dependent and independent stomatal closure, and delayed stomata re‐opening during drought recovery (the ‘after‐effect’ of drought, a feature of drought stress memory). They also contribute to drought‐triggered leaf epidermis and cuticle changes, for which the main driver is however the KAI2 ligand/karrikin pathway in arabidopsis. Finally, they can ensure the sustainability of a water‐spending approach by favouring arbuscular mycorrhizal (AM) symbiosis and biomass allocation to the roots, thus allowing better soil exploration and water capture. [Color figure can be viewed at wileyonlinelibrary.com]

Initial reports pointed out how strigolactone‐insensitive or depleted lines of arabidopsis (Ha et al., 2014), *Lotus japonicus* (lotus) (J. Liu et al., 2015), tomato (Visentin et al., 2016) and barley (Marzec et al., 2020) are hypersensitive to osmotic stress and wilt more easily than their wild‐type counterparts when water becomes scarce. Note that in plants, osmotic stress is usually triggered by reduced irrigation and/or high salinity, but also—namely, under laboratory conditions—by the presence of osmotically active compounds such as mannitol or polyethylenglycol (PEG). The above hyper‐sensitivity is generally linked to lower relative water content, impaired photosynthesis, and higher stomatal conductance, coupled to sometimes altered stomatal density, larger stomata and/or slower stomata closure in response to drought. This latter feature in turn has been linked to defects in abscisic acid (ABA) sensitivity and, less consistently, ABA synthesis (Figure 2) (reviewed in Cardinale et al., 2018).

Higher stomatal conductance for similar leaf water potential is more obvious under irrigated and mild stress conditions, while under severe stress, hydraulic signals seem to prevail and no differences are often seen in stomatal conductance of strigolactone‐related mutants and wild‐type (Visentin et al., 2016). In accordance with the above features, transcriptomics on *d14* mutants point to a wide dysregulation of stress‐ and ABA‐responsive genes in arabidopsis plants undergoing drought, even though an ABA‐independent signature is detectable (W. Li, Nguyen, Chu, et al., 2020). Rice seems to be the only exception in this picture, since mutants lacking proteins downstream of D27 in the biosynthetic pathway are reported as more drought tolerant than the wild‐type, due to the—so far unique—negative correlation between strigolactone and ABA contents in this species (Haider et al., 2018) that was confirmed later (X. Liu et al., 2020). What the significance of such specific difference may be in rice ecology awaits investigation.

Consistent with the hypersensitive phenotype of the strigolactone mutants, drought induces the transcription of strigolactone biosynthetic genes in the leaves of all tested species, such as arabidopsis and tomato (Ha et al., 2014; Visentin et al., 2016; Visentin et al., 2020)—although it has so far proven impossible to detect the metabolites in aerial tissues. This is likely due to a technical sensitivity issue: even in the presence of drought‐induced activation, the transcripts of the biosynthetic genes are about 100‐fold less concentrated in the leaves than in unstressed root tissues (Visentin et al., 2016). Furthermore, biosynthesis may be localized to only certain cell types, leading to dilution and/or the production of strigolactone variants of undescribed structure that will go undetected. The strigolactone scientific community still misses the broad availability of a genetically encoded strigolactone sensor or reporter, allowing the direct visualization of sites and intensities of strigolactone action at the cell level *in planta*. Some attempts have been made in this direction (Samodelov et al., 2016; Sanchez et al., 2018; Song et al., 2022), all relying on the quantification of fluorescent versions of either the D14 or SMXL6 protein. While D14 degradation upon strigolactone perception is slower than that SMXL6, the latter may not be the (only) transducer that is degraded in a specific cell type and process and thus, (part of) the strigolactone signal may be missed. For example, SMAX1, SMXL7/8 or BES1 might take substantial part in transduction cascade downstream of D14, to cite only the most obvious (see above). Especially Strigo‐D2 seems a promising tool nonetheless, because cell‐level definition in live tissues appears good and calibration reliable (Song et al., 2022). However, it still needs to be tested under stress conditions.

It is to be noted that under osmotic stress, the concentration of strigolactone in non‐mycorrhizal roots shows a somewhat counterintuitive decrease (Aroca et al., 2013; Haider et al., 2018; J. Liu et al., 2015; Min et al., 2019; Ruiz‐Lozano et al., 2016; Visentin et al., 2016). This decrease is needed for local ABA rise in lotus (J. Liu et al., 2015), and sufficient for the activation of the strigolactone biosynthetic pathway in the leaves of tomato (Visentin et al., 2016). Thus, a model has been put forward in tomato whereby the drop in root‐synthesized strigolactones is sensed by the shoot, where it triggers a local increase in strigolactone synthesis, for improved drought acclimation (Cardinale et al., 2018; Visentin et al., 2016). It appears also that this organ‐specific biosynthetic pattern of strigolactones is unique for osmotic stress, as other cues that may carry an osmotic component, such as heat stress, induce an increase of strigolactones in the roots (Chi et al., 2021). It is notable, and calls for more investigations, that the issue has not yet been addressed in arabidopsis.

How systemic sensing happens is also interesting and still unresolved. It may happen indirectly, with the strigolactone decrease in the roots triggering an as yet undefined shootward signalling pathway of which the mediator(s) are unrelated to strigolactones. However, given the demonstrated upward mobility of strigolactone themselves in the xylem parenchyma, and their ability to repress their own biosynthetic pathway, it may also occur directly, with the loss of root‐derived strigolactones de‐repressing synthesis in the shoot under drought (Cardinale et al., 2018). This is a key point that needs further investigation.

Additionally, with all the caveats described above about the use of *rac*‐GR24 versus its more specific enantiomers (Figure 1 right), short‐term stomatal closure in the absence of stress has been observed in a number of species upon GR24 treatment, among which arabidopsis (Lv et al., 2018), faba bean (*Vicia faba* L.) (Y. Zhang et al., 2018) and tomato (Visentin et al., 2016). This was shown to be ABA‐independent and dependent on hydrogen peroxide (H_2_O_2_) and nitric oxide production in arabidopsis (Lv et al., 2018). Given the effect of strigolactones on hydrogen sulphide synthesis (Huang et al., 2021) and the role of the latter in stomatal closure downstream of H_2_O_2_ (Liu & Xue, 2021), a possible involvement of hydrogen sulphide in strigolactone‐triggered stomatal closure should be investigated. Thus, it appears that a strigolactone‐related genetic defect sets a higher steady‐state of stomatal conductance via both ABA‐dependent and ABA‐independent paths. On the other hand, the short‐term effects on stomata closure by the synthetic analogue GR24 may be fully ABA‐independent, even though the issue could not be investigated further in other plant species for lack of the appropriate mutants (for a detailed description of this model and of the systemic versus local signalling it entails, see Cardinale et al., 2018).

Finally, it must be also noted that mycorrhization and increased root:shoot biomass ratio are also considered ways to avoid not only nutrient‐ but also water‐related stress, namely in water spenders. Thus, the promotion of mycorrhization (not treated here, see Lanfranco et al., 2018) and of root biomass allocation (see below) by strigolactones can be seen as yet another aspect of their positive influence on stress avoidance.

### 3.2 Strigolactones and environmental memory: A role as regulators of priming for drought avoidance

Two recent papers describe the effects of strigolactones on the memory of a recent drought spell, whereby stomatal opening is significantly delayed after water potential has recovered. This hysteresis, promoted by ABA, goes under the name of ‘after‐effect’ and was shown to be promoted by strigolactones in arabidopsis (Korwin Krukowski et al., 2022) and tomato (Visentin et al., 2020). In fact, low strigolactone content or sensitivity impair the after‐effect in both species, while treatment of wild‐type tomato plants with GR24^5DS^ 24 h before dehydration prolongs it.

Both unbiased and targeted hypotheses have led to the identification of strigolactone transduction components affecting ABA sensitivity and activity, and the after‐effect more in general. In arabidopsis, in silico analyses on the transcriptome of wild‐type and *d14* plants during drought and recovery have highlighted that about two‐thirds of the genes induced by stress show prolonged or exclusive activation through recovery, and that about three‐fourths of them depend on strigolactones to do so. It has also singled out a few prominent potential transducers of strigolactone‐dependent transcriptional regulation after stress, among which BES1. The importance of strigolactone‐triggered degradation of BES1 for a correct transcriptional response to drought and recovery has been cleanly demonstrated (Korwin Krukowski et al., 2022). This study followed the expression of target ‘memory’ genes during a full stress time‐course in the *bes1‐d* mutant, which carries a gain‐of‐function allele insensitive to MAX2 action. Given the participation of BES1 in multiple signalling networks, this set‐up allowed the authors to uncover all MAX_2_^‐^dependent effects of BES1 on recovery from drought stress, without the confounding effects of a complete *BES1* knock‐out.

In tomato, the conserved microRNA miR156 is dependent on strigolactones for drought stress induction, and miR156 overexpression intensifies the after‐effect of drought also by increasing ABA sensitivity at the guard cell level, but not total leaf ABA content (Visentin et al., 2020). The more‐than‐additive synergy between strigolactone treatment and drought in miR156 induction during recovery is remarkable (Visentin et al., 2020), and points to a possible effect of GR24^5DS^ in priming for drought responses at the level of mature miR156 production. Whether this priming is also detectable in repeated drought stresses either at the miR156 or stomatal level, is still an open question. It is worth noting, however, that many arabidopsis genes, of which the expression profile is strigolactone‐dependent in the recovery phase after drought, were also defined as ‘memory genes’ for repeated dehydration stress in previous works (Ding et al., 2013; Hopper et al., 2014; Korwin Krukowski et al., 2022; Virlouvet & Fromm, 2015). Also, miR156 is important for the memory of repeated heat stress in arabidopsis (Stief et al., 2014) and the overlap in strigolactone functions during drought and heat stress extends beyond miR156. For example, strigolactones are crucial for the transcriptional induction of heat shock factors and proteins, which in turn are important for heat stress resistance together with BES1 (Albertos et al., 2022), as well as for the transcriptional memory of stress and priming towards acclimation (Chi et al., 2021; Ha et al., 2014; Korwin Krukowski et al., 2022; H.‐C. Liu et al., 2018; L. Wang, Wang, et al., 2020). These findings collectively suggest that strigolactones may be connected to environmental memory, acting to set the plant alert level with respect to a fluctuating environment**—**namely for drought avoidance. As a general consensus exists on the role of chromatin modifications in the generation of stress memory (Bäurle, 2018), this opens the yet unexplored possibility that strigolactones may regulate the chromatin status. More experiments devised to directly target this hypothesis and its molecular bases are needed.

## 4 STRIGOLACTONES AS REGULATORS OF STRESS TOLERANCE

**Figure 3:**
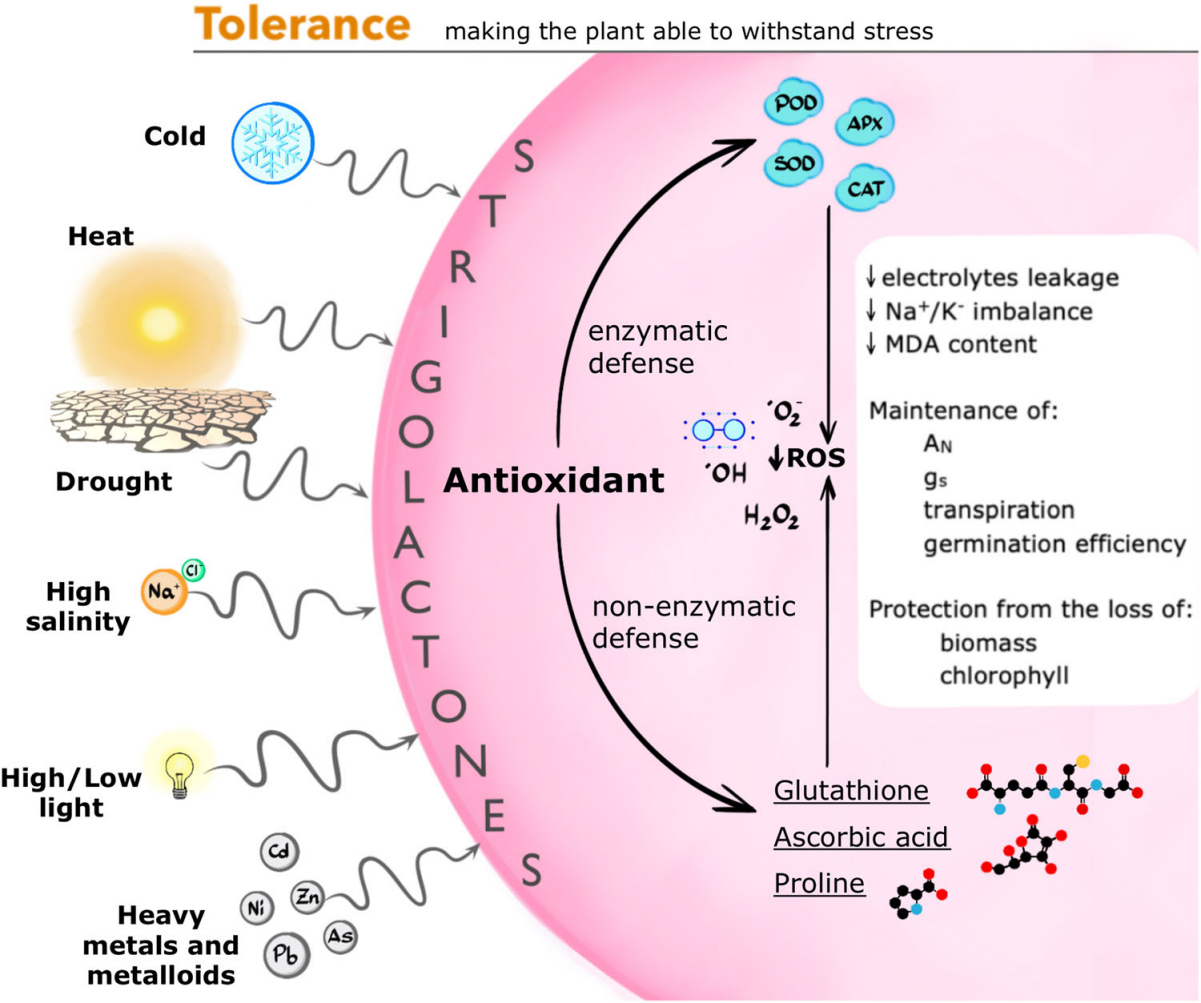
The influence of strigolactones (SLs) on environmental stress tolerance. Strigolactones support the biochemical mitigation of stress effects by promoting enzymatic and non‐enzymatic antioxidants. Given the cross‐cutting occurrence of reactive oxygen species (ROS) in a variety of stressful conditions, this effect has been recorded so far in conditions of drought, high osmolarity/salinity, high/low light, heat, cold, and heavy metal/metalloid soil contamination. A_N_, net assimilation; APX, ASCORBATE PEROXIDASE; CAT, CATALASE; H_2_O_2_, hydrogen peroxide; g_s_, stomatal conductance; MDA, malondialdehyde; POD, PEROXIDASE; SOD, SUPEROXIDE DISMUTASE. [Color figure can be viewed at wileyonlinelibrary.com]

Over the past few years, the inability of plants lacking strigolactones or their sensing to mount normal mitigation responses to a range of abiotic stresses has also emerged. These responses aim to reduce the impact of osmotic or metabolic imbalances on cellular activities, and especially the accumulation and negative effects of reactive oxygen species (ROS) (Figure 3). Given the cross‐cutting role of ROS as effectors of cell damage during all kinds of suboptimal conditions, it is not surprising that molecules able to mitigate their effects can be beneficial across different stresses. Consistently, a trove of findings with potential application impact has been building up, especially in connection with the use of GR24 treatment before or during stress to improve crop performances. The stresses covered by this kind of studies range from heat and cold (Chi et al., 2021; Omoarelojie et al., 2020, 2021, X. Zhang et al., 2020); low light, and related senescence and growth inhibition (Lu et al., 2019; Mayzlish‐Gati et al., 2010; Tian et al., 2018; Zhou et al., 2022); high light (Thula et al., 2022); to heavy metals such as cadmium (Chen et al., 2007; Niu et al., 2021; Qiu et al., 2021; Tai et al., 2017) and the metalloid arsenic (Mostofa, Ha, et al., 2021; Mostofa, Rahman, et al., 2021). We will focus here mostly on the connections between strigolactones and responses to osmotic stress, while the results exploring protection from other abiotic stresses are summarized in Table 1. It should be highlighted that with a few exceptions, the published reports on GR24 treatments carry the confounding effects of the racemic mixture and the plausible stimulation, in parallel, of both the D14 and KAI2‐dependent pathways (see above). This point is relevant here and highlighted by each table item, as it appears that also karrikins and KAI2‐dependent responses can help tolerate abiotic stresses (Ahmad et al., 2021; W. Li, Gupta, et al., 2020; W. Li et al., 2017) and that additional molecular cross‐talk between the two signalling pathways may occur in the context of stress (see below).

**Table 1:** Synopsis of selected articles in which evidence for strigolactone‐related tolerance of environmental stresses is reported

Type of stress	GR24 treatment	Plant species	Mutant(s)	Biometrics	Physiology	Gene expression	References	
Low light	GR24 spray treatment^a^	*Solanum lycopersicum*	‐	+ Shoot and root DW + Root FW − Plant height	+ Chlorophyll and photosynthetic rate + PSII activity + SOD and POD activity − MDA and H_2_O_2_	+ Expression of genes encoding core proteins of the PSII and PSI reaction centre + *SOD* and *POD* gene expression	Lu et al. (2019)	
	GR24 spray treatment^a^	*Cucumis sativus*	‐	+ Shoot and root FW and DW + Leaf length, width and total area + Stem diameter	+ Chlorophyll and photosynthetic rate + Transpiration and stomatal conductance + Total soluble sugar and sucrose + Antioxidants − MDA and H_2_O_2_	+ Expression of genes for antioxidant (*APX*, *GR*, *DHAR* and *MDHAR*) and sucrose metabolism enzymes − *RBOH* expression	Zhou et al. (2022)	
High light	*rac‐*GR24 in agarised medium	*Arabidopsis thaliana*	*hpe1‐1, hpe1‐2, d14‐1, htl3*	‐	+ Modulation of metabolite flux (sugar and derivatives, intermediate component and amino acids). − Chlorophyll and carotenoids	− *APX6*, *CAT2*, *SOD2* expression + *HSP* and anthocyanin‐related gene expression *(MYB114, PAP2*)^b^	Thula et al. (2022)	
Cd	GR24 in nutrient solution^a,c^	*Panicum virgatum*	‐	+ Shoot and root DW	+ Root RWC + Chlorophyll and photosynthetic rate + Transpiration and stomatal conductance + SOD and CAT activity − MDA, Cd accumulation	‐	Tai et al. (2017)	
	*rac*‐GR24 in nutrient solution^c^	*Hordeum vulgare*	‐	+ Plant height + Root length + Shoot and root DW	+ Nutrient uptake + NOS, DHAR, MDHAR, APX, GPX, GR, CAT, SOD and POD activity + Non‐enzymatic scavenging and NO − MDA and H_2_O_2_, Cd accumulation	‐	Qiu et al. (2021)	
Na_3_AsO_4_ c	‐	*Oryza sativa*	*d10* and *d17*	+ Primary root length + Shoot height + Shoot and root DW	+ Shoot RWC, nutrient uptake + Chlorophyll and carotenoids + SOD, CAT and APX activity, GSH − Electrolyte leakage, MDA and H_2_O_2_, root As accumulation	+ *SOD*, *CAT* and *APX* expression + Phytochelatin and GSH‐related gene expression + Expression of Pi transporter‐encoding genes	Mostofa, Ha, et al. (2021); Mostofa, Rahman, et al. (2021)	
Low T	GR24 spray treatment^a^	*Brassica rapa*	‐	‐	− MDA and H_2_O_2_ + Soluble protein and proline + SOD, CAT, POD and APX activity	+ *ICE1*, *COR1*, *MAPKs*, *SOD*, *CAT*, *POD* and *APX* expression − *RBOHs* expression	X. Zhang et al. (2020)	
	*rac*‐GR24 in irrigation solution	*Vigna radiata*	‐	‐	+ RWC + Total soluble sugar and proline + PAL, TAL, SOD and LOX activity − O_2_^−^, H_2_O_2_, phenolics and MDA	‐	Omoarelojie et al. (2021)	
High T	*rac‐*GR24 in nutrient solution	*Lupinus angustifolius*	‐	+ Seed germination	+ Proline + GlyI, GlyII, SOD, POD and APX activity − MDA	‐	Omoarelojie et al. (2020)	
High and low T	GR24^5DS^ in irrigation solution	*S. lycopersicum*	*ccd7*, *ccd8*, *max1*, *max2*	‐	+ SOD, APX, GR, MDAR and DHAR activity + Leaf ABA + HSP70 and CBF1	+ *HSP70* and *CBF1* expression + *NCED6* expression	Chi et al. (2021)	
Osmotic stress (mannitol/NaCl/drought)		*Sapium sebiferum*, *A. thaliana*	*SsMAX2* overexpressing line and *max2*	+ Seed germination − Branch number − Hypocotyl length	+ Chlorophyll and anthocyanin + Proline and soluble sugar + CAT, SOD and POD activity − Water loss, MDA and H_2_O_2_	+ Expression of anthocyanin biosynthetic genes (*CHS, CHI, F3H, F3'H, DFR* and *ANS*)	Q. Wang et al. (2019)	
NaHCO_3_ and NaCl	GR24 spray treatment^a^	*Malus* *hupehensis*	‐	+ Total DW and FW	+ Proline + Chlorophyll and photosynthetic rate +Transpiration and stomatal conductance + Nutrient uptake + IAA, GA3, ZR and JA + SOD POD and CAT activity − H_2_O_2_, O_2_^−^, OH* and MDA − Electrolyte leakage and Na/K ratio − Soluble protein and wilting rate	+ H^+^ ATPase gene expression + *SOD*, *POD* and *CAT* expression + *MAX2*, *D14*, *CCD7*, *CCD8*, *D27, D53* and *CYP711* expression^d^ + Na^+^ transporter‐encoding genes − K^+^ transporter‐encoding genes	C. Ma et al. (2022)	
NaCl	GR24 in soaking solution (seeds)^a^	*Triticum aestivum*	‐	+ Shoot length + Root FW	− Stomatal conductance + Photosynthetic rate	‐	Kausar and Shahbaz (2017)	
	GR24 in nutrient solution^a^	*Brassica napus*	‐	+ Root and shoot FW and DW	+ Chlorophyll and photosynthetic rate + Transpiration and stomatal conductance + SOD and POD activity − MDA	‐	N. Ma et al. (2017)	
	GR24 in nutrient solution^a^	*O. sativa*	‐	+ Plant height + Root length + Root and shoot DW	+ Chlorophyll and photosynthetic rate + Transpiration and stomatal conductance + POD and SOD activity − MDA	‐	Ling et al. (2020)	
	GR24 in nutrient solution^a^	*S. lycopersicum*	‐	+ Leaf area + Root length	+ Chlorophyll and carotenoid + CAT, SOD, POD, APX and GR activity − AsA	+ *CCD7*, *CCD8*, *D14, D27, MAX1* and *MAX2* expression^d^	H. Liu, Able, et al. (2022); H. Liu, Li, et al. (2022)	
	GR24 spray treatment^a^	*Helianthus annuus*	‐	+ Shoot length + Shoot and root (or callus) DW and FW	+ Chlorophyll and carotenoid + Transpiration rate + Leaf water potential and RWC + Osmotic and turgor potential + Root and shoot K^+^ and Ca^2^ − Root and shoot Na^+^	‐	Sarwar and Shahbaz (2019, 2020); Zulfiqar et al. (2021)	
	GR24 in soaking solution (achenes)^a^							
	GR24 in agarised medium (calli)^a^							
	GR24 spray treatment^a^	*Salvia nemorosa*	‐	+ Plant growth rate	+ Chlorophyll and photosynthetic rate + Transpiration and stomatal conductance + Proline and total phenolics + POD, SOD, CAT and GR activity + Essential oil yield and content − Electrolyte leakage, H_2_O_2_ and MDA	‐	Sharifi and Bidabadi (2020)	
	GR24 spray treatment^a^	*C. sativus*	‐	‐	+ Proline + SOD, POD, CAT and APX activity − Electrolyte leakage, O_2_^−^, OH* and H_2_O_2_	+ *SOD*, *POD*, *CAT* and *APX* expression − *RBOH* expression	X.‐H. Zhang et al. (2022)	
	GR24 spray treatment^a^	*Zea mays*	‐	+ Main cob diameter, no. of grains/cob	+ Chlorophyll, carotenoids and photosynthetic rate + Transpiration and stomatal conductance + K^+^ − Na^+^	‐	Iftikhar et al. (2022)	
KCl	GR24 spray treatment^a^	*M. hupehensis*	‐	+ Plant height + Seedling FW and DW	+ Chlorophyll and photosynthetic rate + Transpiration and stomatal conductance + POD and CAT activity + Proline, nutrient uptake − SOD activity, wilting − H_2_O_2_, O_2_^−^, OH* and MDA	‐	Zheng et al. (2021)	
Drought	*rac*‐GR24 spray treatment^c^	*Vitis vinifera*	‐	+ Stomatal density − Stomatal length, width and opening	+ RWC and chlorophyll + Root MeJA − SOD, POD, CAT, APX activity − Leaf and root ZR and ABA, leaf MeJA − Electrolyte leakage, GSH, AsA, MDA and H_2_O_2_	+ *NCED1*, *HYD1*, *HYD2* and *BRC1* expression^b^	Min et al. (2019)	
	GR24 spray treatment^a^	*T. aestivum*	‐	+ Root and shoot DW + Root biomass	+ RWC + Photosynthetic rate + Transpiration and stomatal conductance + SOD, POD, CAT and APX activity − H_2_O_2_	‐	Sedaghat et al. (2021)	
	*rac*‐GR24 in irrigation water or spray treatment^c^							
	GR24 spray treatment^a^	*Medicago sativa*	‐	+ Root and shoot length + Root, stem and leaf FW	+ Leaves and roots soluble protein content + POD and CAT leaves activity + POD, CAT and SOD root activity	+*PYL4*, *PYL8*, *PP2C1*, *PP2C50* (under stress), *ABI5‐like2*, *bZIP46*, *BCAT2*, *ASN*, *SMAX1* − *PP2C24* and *PP2C50* (under no stress), *KAI2*, *MAX2*	Y. Yang et al. (2022)	

*Note*: The (+) and (−) marks before different variables indicate a positive or negative correlation with strigolactone levels, either exogenous (as GR24) and/or endogenous (in the comparison between wild‐type and strigolactone‐related mutants). The correlation may be observed under stress and/or normal conditions.

Abbreviations: ABA, abscisic acid; ABI, ABA‐INSENSITIVE; ANS, ANTHOCYANIN SYNTHASE; APX, ASCORBATE PEROXIDASE; AsA, ascorbic acid; ASN, ASPARAGINE SYNTHETASE; BCAT, BRANCHED‐CHAIN‐AMINO‐ACID AMINOTRANSFERASE; BRC, BRANCHED; bZIP, BASIC REGION‐LEUCINE ZIPPER; CAT, CATALASE; CBF1, C‐REPEAT BINDING FACTOR1; CCD, CAROTENOID CLEAVAGE DIOXYGENASE; CHI, CHALCONE‐FLAVANONE ISOMERASE; CHS, CHALCONE SYNTHASE; COR, CRT (C‐REPEAT)/DRE; DMAR, dry matter accumulation rate; D, DWARF; DFR, DIHYDROFLAVONOL REDUCTASE; DW, dry weight; FW, fresh weight; F3H, FLAVANONE 3‐HYDROXYLASE; F3'H, FLAVANONE 30‐HYDROXYLASE; GA3, gibberellic acid; GlyI, II, GLYOXALASEI, II; GPX, GLUTATHIONE PEROXIDASE; GR, GLUTATHIONE REDUCTASE; GSH, reduced gluthatione; HPE, HIGH PHOTOSYNTHESIS EFFICIENCY; HSP70, HEAT SHOCK PROTEIN70; HYD, ABA 8'‐HYDROXYLASES; KAI2, KARRIKIN INSENSITIVE2; IAA, indol‐3 acetic acid; ICE1, INDUCER OF CBF EXPRESSION1; JA, jasmonic acid; LOX, LIPOXYGENASE; MAPK, MITOGEN‐ACTIVATED PROTEIN KINASE; MAX, MORE AXILLARY GROWTH; MDA, malondialdehyde; MDHAR, MONODEHYDROASCORBATE REDUCTASE; MeJA, methyl JA; MYB, MYELOBLASTOSIS; NCED, NINE‐CIS‐EPOXYCAROTENOID DIOXIGENASE; NOS, NO synthetase; PAL, PHENYLALANINE AMMONIA‐LYASE; PAP, PRODUCTION OF ANTHOCYANIN PIGMENT; POD, PEROXIDE DISMUTASE; PP2C, PROTEIN PHOSPHATASE 2C; PS, photosystem; PYL, PYRABACTIN‐RESISTANCE1‐LIKE; SL, strigolactone; RBOH, RESPIRATORY BURST OXIDASE HOMOLOGUES; RWC, relative water content; SOD, SUPEROXIDE DISMUTASE; SMAX1, SUPPRESSOR OF MAX2 1; TAL, TYROSINE AMMONIA‐LYASE; ZR, zeatine riboside.

^a^ Indicates that in lack of clear indications in the article, *rac*‐GR24 should be assumed to have been used; thus, results likely reflect the activation of both D14‐ and KAI2‐dependent pathways.

^b^ Transcriptomics of *rac*‐GR24 or GR24^5DS^‐treated tissues.

^c^ A mixed stress‐coping strategy (avoidance and tolerance) seems induced by GR24 (e.g., by increasing root/shoot biomass ratios or reducing the uptake of soil pollutants).

^d^ The experiment lacks controls with GR24 treatment alone, so the correlation of strigolactone levels with gene expression is only reported in the presence of stress.

### 4.1 Induction of tolerance to drought and salinity stress

The effects of GR24 treatments on responses to high salinity were tested in several species: arabidopsis (Ha et al., 2014); beet (*Brassica napus* L.) (N. Ma et al., 2017); rice (Ling et al., 2020); cucumber (*Cucumis sativus* L.) (X.‐H. Zhang et al., 2022); tomato (H. Liu, Li, et al., 2022); wheat (*Triticum aestivum* L.) (Kausar & Shahbaz, 2017); sunflower (*Helianthus annuus* L.) (Sarwar & Shahbaz, 2019, 2020; Zulfiqar et al., 2021); wild sage (*Salvia nemorosa* L.) (Sharifi & Bidabadi, 2020); corn (*Zea mays*) (Iftikhar et al., 2022); and apple tree (*Malus hupehensis* Rehd) (C. Ma et al., 2022; Zheng et al., 2021). A realignment of physiological and molecular indicators to unstressed controls was consistently observed following treatment, namely in terms of protection from a drop in germination efficiency, biomass and/or chlorophyll content loss; maintenance of gas exchange parameters (net photosynthetic rate, stomatal conductance, transpiration); and lower malondialdehyde content (MDA, a biomarker of polyunsaturated lipid peroxidation that accumulates in stressed cells). This is most often accompanied, and likely explained at least in part, by a general increase in the activity of antioxidant enzymes such as peroxide dismutase (POD), superoxide dismutase (SOD), ascorbate peroxidase (APX) and/or catalase (CAT), along with non‐enzymatic antioxidant defences. When measured, ROS content generally increases less in the presence of GR24 during stress than during stress alone, while electrolyte leakage and Na^+^/K^+^ imbalance are reduced. Treatment with DPI (diphenyleneiodonium chloride, an NADPH oxidase inhibitor), DMTU (dimethylthiourea, an H_2_O_2_ scavenger) or EGTA (ethylene glycol tetraacetic acid, a Ca^2+^ chelating agent) can reverse the positive effect of *rac‐*GR24 treatment on salt‐stressed plants, as observed in cucumber (X.‐H. Zhang et al., 2022); this supports the view that H_2_O_2_ and Ca^2+^ are both indispensable to GR24 action. Finally, both *rac‐*GR24 and GSNO (the NO donor S‐nitrosoglutathione) have been tested during salt stress in tomato. GSNO has similar effects as *rac*‐GR24 in terms of increased antioxidant enzyme activity and chlorophyll content, mitigating the damage caused by salt exposure. However, in the presence of TIS108 (a triazole‐type strigolactone synthesis inhibitor), the protective role of NO during salt stress is reduced, suggesting that strigolactones are necessary for the NO‐induced protective response to salt stress (H. Liu, Li, et al., 2022). A similar positive correlation between strigolactones and drought tolerance via increased antioxidant activity was highlighted in winter wheat (Sedaghat et al., 2017, 2021) and *Medicago sativa* (Y. Yang et al., 2022).

A few studies make use of genotypes altered in the synthesis or perception of strigolactones to draw conclusions on their role in stress mitigation. The overexpression in arabidopsis of the *MAX2* homolog *SsMAX2* from the popcorn tree *Sapium sebiferum* confers resistance to osmotic stress, and increases proline and soluble sugar content as well as CAT, SOD and POD activity when compared to the wild‐type. As a likely consequence, H_2_O_2_ and MDA concentrations are lower in the *SsMAX2*‐overexpressing lines, while the *max2* mutant has the opposite molecular and physiological phenotype (Q. Wang et al., 2019). Of course, these results could be ascribed to the D14‐dependent pathway but also, as not emphasized enough by the authors, to the KAI2‐dependent one (see dedicated paragraph below). Nonetheless, the salinity‐sensitive phenotype of strigolactone‐only mutants, such as *max3* and *max4*, is documented (Ha et al., 2014), so the pathway contribution in the phenotype of *SsMAX2*‐overexpressing arabidopsis remains likely. Also, the role of *MAX2*, *MAX3* and *MAX4* in drought resistance was originally assessed by the use of mutants (Ha et al., 2014). Consistently, the triple mutant *smxl6/7/8*, more than the single and double mutants of these repressors of strigolactone action, has a better survival rate after drought than the wild type and, of course, than the *max2* mutant (T. Yang et al., 2020). In this picture, the only exception seems to be rice, for which—as mentioned above—all strigolactone‐related mutants but *d27* are more drought‐tolerant than the wild type (Haider et al., 2018).

### 4.2 The acclimation dilemma: To avoid stress, to tolerate it, or both?

In winter wheat subjected to drought stress, treatment with *rac*‐GR24 and/or salicylic acid increases membrane stability and the activity of POD, CAT, APX, SOD—especially when combined. For some of the tested parameters, the hormonal treatments are beneficial also on irrigated plants, which display lower MDA content and electrolyte leakage, and higher APX activity with respect to the untreated, unstressed plants (Sedaghat et al., 2017). Notably, a strigolactone‐salicylic acid crosstalk has been recently suggested in the frame of biotic stress responses as well (Kusajima et al., 2022). Sedaghat et al. (2017) have also ascertained that winter wheat performances under drought are improved to slightly different extents depending on whether GR24 is applied on the roots or leaves, and also that root application is especially effective at increasing root biomass. This in itself can justify more efficient water capture by treated plants, which can afford to keep stomata more open and sustain photosynthesis better than untreated, stressed plants. In the case of GR24‐treated winter wheat under drought, we thus observe both better tolerance via increased antioxidant defences (SOD, CAT, POD, APX activity), and the parallel adoption of a water‐spending strategy (in itself, a feature of drought avoidance) (Sedaghat et al., 2021).

The increase in root biomass following GR24 treatment was also observed in other plants su tomato and switchgrass (*Panicum virgatum* L.) (Tai et al., 2017; Santoro et al., 2020); consistently, strigolactone‐depleted genotypes tend to have lower root/shoot biomass ratios, although with exceptions (Rasmussen et al., 2013). In a further example, cadmium stress is both better tolerated in GR24‐treated plants, and in part avoided, since it appears that treatment helps the plant exclude the toxic metal, reducing its uptake (Qiu et al., 2021; Tai et al., 2017). A similar situation is reported for arsenate (Mostofa, Ha, et al., 2021; Mostofa, Rahman, et al., 2021). Finally, *rac*‐GR24 triggers a more efficient water‐saving phenotype (and thus, drought avoidance) in leaves of grapevine (*Vitis vinifera* L.) under PEG‐induced osmotic stress, by allowing to maintain net carbon assimilation rates in the presence of lower stomatal conductance. GR24 treatment is also associated to lower electrolyte leakage and increased water and chlorophyll content compared to the non‐treated, stressed plants, which may be seen as both a feature of increased stress tolerance, and of avoidance. In this experimental set‐up, though, the enzymatic and non‐enzymatic antioxidant system was less activated and yet, the H_2_O_2_ and MDA content were lower in GR24‐treated plants. This suggests that stress was rather avoided than better tolerated; however, the work lacks appropriate controls on GR24‐treated, unstressed plants (Min et al., 2019).

To conclude, it is important to keep in mind that depending on the plant species, the stress conditions and the mode/timing/dose of administration, GR24 may push the plant to mostly avoid rather than mitigate osmotic stress consequences, or vice versa; or yet, to do both. It is possible, for example, that if stress occurs fast and is severe, tolerance will not have time to become effective; and a stress avoidance strategy may rather be deployed. It is known indeed that different stress application protocols can lead to different outcomes and physiological responses, for example in woody plants (Morabito et al., 2022; Ruehr et al., 2019). In tomato, GR24^5DS^ delivered before abrupt dehydration clearly instigates a water‐saving strategy (Visentin et al., 2020), while under different conditions, *rac*‐GR24 induces tolerance to heat and cold stress (Chi et al., 2021). Thus, an interesting and still pending question is whether strigolactones may be important for both or either strategies in the same plant species, subjected to a more or less sudden or intense water deprivation stress (e.g., abrupt dehydration vs. gradual water withdrawal), for which targeted experiments should be devised.

### 4.3 Interactions between the D14‐ and KAI2‐dependent pathways in drought tolerance and avoidance

As mentioned above, the KAI2 pathway is involved in drought stress responses along with D14 (W. Li et al., 2017; W. Li, Nguyen, Chu, et al., 2020) as well as in other abiotic stresses (T. Yang et al., 2019). While the general processes affected by the two pathways largely overlap in relation to drought, there are some specificities at a more granular level. For example, KAI2 seems to have a stronger role in the maintenance of cell membrane integrity, leaf cuticle structure and ABA‐induced leaf senescence than D14, but a weaker role in drought‐induced anthocyanin biosynthesis and effect on brassinosteroid and cytokinin signalling. Instead, both pathways affect photosynthesis and the metabolism of glucosinolates and trehalose, and an additive effect is suspected in regulating cell membrane integrity and leaf cuticle development (W. Li, Gupta, et al., 2020; W. Li, Nguyen, Chu, et al., 2020). It is also interesting to notice that even within the same biological process, different genes may depend from either pathway for proper regulation under drought. For example, specific gene subsets in the ABA‐responsive category are misregulated in the *kai2* or *d14* mutants under drought stress (W. Li, Gupta, et al., 2020; W. Li, Nguyen, Chu, et al., 2020). The only partially redundant action of D14 and KAI2, and the fact that both signalling pathways converge at MAX2, justifies the fact that the hypersensitivity to osmotic stress of *max2* or *d14kai2* plants is more severe than either *kai2* or *d14* individual mutants (Bu et al., 2014; W. Li, Nguyen, Chu, et al., 2020).

Furthermore, other components downstream of D14 and KAI2 may be shared under stress. By limiting the observation to drought/osmotic stress only, SMXL6/7/8 have been identified as the repressors of drought responses that are brought to degradation by interaction with MAX2 (W. Li, Nguyen, Tran, et al., 2020; T. Yang et al., 2020). However, a recent paper has shown that it is SMAX1 to be degraded in dependence of D14 and MAX2 action under osmotic stress, as mentioned in § 2.2 (Q. Li et al., 2022). Thus, here again, the fact that the strigolactone and KAI2 ligand/karrikin‐triggered transduction pathways are not insulated makes it important to confirm experimentally both in silico predictions and assumptions based on previous findings obtained in different conditions, thus correctly identifying what components are involved in each specific process (in this case, osmotic stress responses).

## 5 STRIGOLACTONES AS REGULATORS OF STRESS ESCAPE: A POSSIBILITY WORTH TESTING

A missing part of the picture is whether strigolactones may also regulate stress escape by affecting the timing of flowering. While direct evidence is sorely needed, several clues suggest that they might, especially in certain plant species. First, strigolactones affect sensitivity to ABA, a hormone that has been proven important for drought escape (Riboni et al., 2016). Second, strigolactone‐related mutants in several (but not all) plant species have reproductive defects. For example, knocking down the biosynthetic gene *CCD7* makes lotus produce fewer flowers, fruits and seeds (J. Liu et al., 2013). Among solanaceous plants, the most severely affected potato (*Solanum tuberosum* L.) lines silenced for *CCD8* do not flower at all (Pasare et al., 2013); and in petunia, delayed flowering time and smaller flowers have been reported for analogous lines (Snowden et al., 2005). In tomato, *CCD8* silencing causes fewer and smaller flowers and fruits (Kohlen et al., 2012); and in pea, *max2* plants have delayed flowering (Rasmussen et al., 2015). So far, little effort has been put into exploring the molecular underpinnings of this phenotype; and even less for a possible intersection between strigolactones, stress and flowering. However, several flowering‐related genes are misregulated in the comparison between wild‐types and strigolactone‐related mutants, both in the absence and in the presence of drought (Ha et al., 2014; Korwin Krukowski et al., 2022). Thus, the next obvious experiments should test the possibility that strigolactones may indeed regulate flowering time in plants experiencing osmotic stress, both in species that do show strigolactone‐related flowering phenotypes under normal conditions—such as solanaceous plants and some leguminous—and species that do not, such as arabidopsis and rice.

## 6 CONCLUSIONS

**Figure 4:**
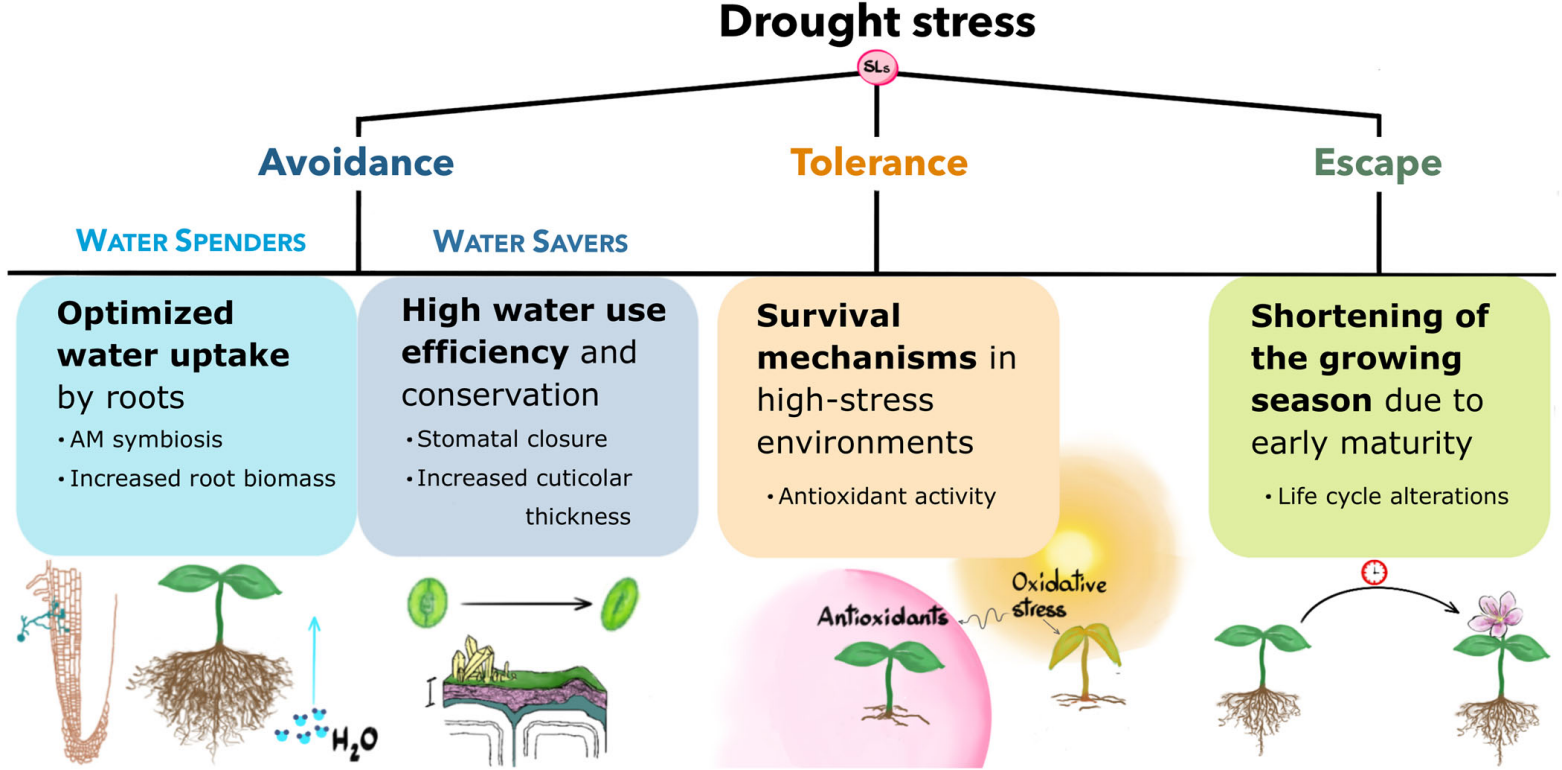
The positioning of strigolactones (SLs) within the main drought stress‐coping strategies: avoidance, tolerance and escape. Several of the pictured concepts apply to other environmental stresses too, as detailed in the text. Their involvement in drought escape is yet to be confirmed. [Color figure can be viewed at wileyonlinelibrary.com]

The number of open questions regarding the functions of strigolactones in stress acclimation are undoubtedly still more numerous than the ones we have answered so far. Nonetheless, it appears clear that strigolactones do play a pivotal role in potentially all main codified aspects of the process (Figure 4). It may be argued that this stems from their modulation of ABA sensitivity/synthesis, a hormone that has long occupied a centre stage position in plant stress biology for its pervasive effects on avoidance, tolerance and escape. However, the sheer fact that strigolactone action also displays ABA‐independent features shows their peculiarities. Additionally, their effects in stress memory and priming, although certainly exerted in part via ABA, highlight how strigolactones may be rather setting the level of alert towards fluctuating environmental conditions, than act as direct effectors of stress acclimation responses. As such, their function would be subtler than the one of ABA, but as important for plant resilience in the long term.

## Data Availability

Data sharing is not applicable—no new data generated.
